# Effects of deficient mismatch repair on the prognosis of patients with stage II and stage III colon cancer during different postoperative periods

**DOI:** 10.1186/s12885-022-10266-3

**Published:** 2022-11-10

**Authors:** Chunze Zhang, Yixiang Zhan, Kemin Ni, Zhaoce Liu, Ran Xin, Qiurong Han, Guoxun Li, Hangyu Ping, Yaohong Liu, Xuanzhu Zhao, Wanting Wang, Suying Yan, Jing Sun, Qinghuai Zhang, Guihua Wang, Zili Zhang, Xipeng Zhang, Xia Hu

**Affiliations:** 1 Department of Colorectal Surgery, Tianjin Union Medical Center, Tianjin, 300121 China; 2 Tianjin Institute of Coloproctology, Tianjin, China; 3 The Institute of Translational Medicine, Tianjin Union Medical Center of Nankai University, Tianjin, China; 4 School of Medicine, Nankai University, Tianjin, China; 5 School of Integrative Medicine, Tianjin University of Traditional Chinese Medicine, Tianjin, China; 6 Tongji Hospital, Tongji Medical College, Huazhong University of Science and Technology, Wuhan, China; 7 The Third Central Clinical College of Tianjin Medical University, Tianjin, China; 8 Department of Agriculture Insect, Institute of Plant Protection, Tianjin Academy of Agricultural Sciences, Tianjin, China

**Keywords:** Colon cancer, Adjuvant chemotherapy, Mismatch repair, Multicenter study

## Abstract

**Background:**

We evaluated the prognostic role of deficient mismatch repair (dMMR) systems in stage II and stage III colon cancer patients during different postoperative periods. We also assessed whether patients aged ≥75 could benefit from chemotherapy.

**Methods:**

This retrospective study was conducted across three medical centers in China. Kaplan–Meier survival methods and Cox proportional hazards models were used to evaluate the differences in overall survival (OS) and disease-free survival (DFS) rates. Propensity score matching was performed to reduce imbalances in the baseline characteristics of the patients. Landmark analysis was performed to evaluate the role of dMMR during different postoperative periods.

**Results:**

The median follow-up time for all patients was 45.0 months (25–75 IQR: 38.0–82.5). There was no significant OS (*p =* 0.350) or DFS (*p* = 0.752) benefit associated with dMMR for stage II and III patients during the first postoperative year. However, significant OS (*p* < 0.001) and DFS (*p* < 0.001) benefits were observed from the second postoperative year until the end of follow-up. These differences remained after propensity score matching. Moreover, chemotherapy produced no OS (HR = 0.761, 95% CI: 0.43–1.34, *p* = 0.341) or DFS (HR = 0.98, 95% CI: 0.51–1.88, *p* = 0.961) benefit for patients aged ≥75 years.

**Conclusion:**

The benefits of dMMR in stage III patients were observed from the second postoperative year until the end of follow-up. However, the prognosis of patients with dMMR is not different from that of patients with proficient mismatch repair (pMMR) during the first postoperative year. In addition, elderly patients aged ≥75 years obtained no significant survival benefits from postoperative chemotherapy.

**Supplementary Information:**

The online version contains supplementary material available at 10.1186/s12885-022-10266-3.

## Background

Colorectal cancer (CRC) is the third most common cancer worldwide, represents approximately 10% of all malignancy [1]. In the MOSAIC study, FOLFOX treatment was found to reduce the risk of death by 20% when compared with LV5FU2 at the 10-year follow-up [2]. Colorectal cancer arises through two known independent pathways, chromosomal instability and dMMR [3]. The mismatch repair (MMR) system monitors and corrects erroneous duplications of base pairs in microsatellites, and four proteins—MLHI, MSH2, MSH6 and PMS2—are the main players in this system. The expression of MMR proteins is localized in the nucleus, and loss of expression is illustrated by the absence of nuclear staining in tumor cells, with nuclear staining occurring in normal epithelial cells, infiltrating lymphocytes, and stromal cells surrounding the tumor. This absence of the expression of one or more of these proteins results in a diagnosis of dMMR; otherwise, the patient is regarded as having pMMR.

If the MMR system does not identify and correct these errors, microsatellites may be repeatedly added or deleted, leading to microsatellite mutations in daughter cells [4]. The frequency of mutations due to polymerase errors in cells with dMMR is more than 100 times that of cells with pMMR [5]. In addition, it has been found that dMMR CRC may not respond to 5­fluorouracil­based chemotherapy [6, 7]. Meanwhile, there are a large number of studies on immunotherapy of gastrointestinal tumors [8], and these studies are mostly related to the MMR status of tumors [9]. Checkmate-142 and NCT01876511 clinical trials have proved that dMMR/MSI-H CRC patients can benefit from immunotherapy [10, 11]. On the basis of the compelling data, the FDA granted accelerated approval to pembrolizumab and nivolumab for the second-line treatment of patients with dMMR/MSI-H CRC. Keynote-177, a phase III trial enrolled 307 patients, further support pembrolizumab as an efficacious first-line therapy for metastatic CRC [12].

The prognosis of stage I CRC patients who undergo radical resection is excellent, with a 5-year overall survival of more than 90% [13, 14]. Hence, stage I patients generally do not receive chemotherapy after surgery. Two-thirds of CRC patients are diagnosed with stage II or III disease at the time of detection [15]. Previously published studies have shown that stage II and III colon cancer patients with dMMR have a better prognosis than those with pMMR [16]; however, whether this remains consistent throughout every stage of the disease is still debated. Nonetheless, the importance of the influence of various factors on recovery differs across different periods after surgery. Surgical complications and adverse chemotherapy reactions caused by adjuvant chemotherapy are concentrated within the first postoperative year [17, 18]. Therefore, MMR states may play different roles during different postoperative periods. We explored the influence of MMR status on prognosis in different postoperative periods. We chose stage II and III colon cancer patients as our primary study subjects.

Studies have shown that patients under the age of 75 receive some benefit from chemotherapy [19, 20]. Elderly colon patients (age > 70 years) of age can receive the same benefit from 5-Fu-based adjuvant therapy as their younger counterpart [21]. The conclusion from retrospective population-based cohort studies show that more than 75 years stage III colon cancer still could get benefit of increasing OS and DFS from adjuvant chemotherapy [22, 23]. In addition, addition of oxaliplatin to 5-Fu as adjuvant treatment for high-risk stage II elder show no statistically significant benefit for OS and DFS [24]. These trails all comes from US and several European countries. However, whether Chinese patients aged 75 or older can benefit from adjuvant chemotherapy remains unclear. Moreover, Chinese patients more than 75 years old with stage II or III colon cancer are undertreated with adjuvant chemotherapy [25, 26]. Few aged ≥75 years participate in clinical trials; thus, we selected this special subgroup for separate analysis.

To determine the exact role of dMMR in stage II and III colon cancer patients during different postoperative periods, we established a retrospective study using data obtained from three Chinese medical centers. Many factors influence the effect of chemotherapy on these patients. Thus, determining whether elderly patients benefit from chemotherapy is significant and essential for determining standard adjuvant chemotherapy treatment procedures. We also assessed the impact of other factors on colon cancer patients.

## Methods

### Study population

Eligible patients were ≥ 18 years of age and underwent complete curative surgery from August 2012 to January 2018 for histologically confirmed TNM stage II or III colon cancer. All patient data were obtained from three Chinese medical centers *(Tianjin Union Medical Center, Tongji Hospital, The Third Central Clinical College of Tianjin Medical University*) and staged according to AJCC Cancer Staging Manual, 8th edition guideline. We collected relevant information about each patient, including pathological reports, personal data, chemotherapy regimens, times to relapse or death and causes of death. Tumors invading through the muscularis propria with no positive regional lymph nodes or distant metastasis was defined as stage II. Positivity of one or more regional lymph nodes or tumor deposits in the subserosa, mesentery, or non-peritonealized pericolic tissues without distant metastasis was defined as stage III.

**Fig. 1:**
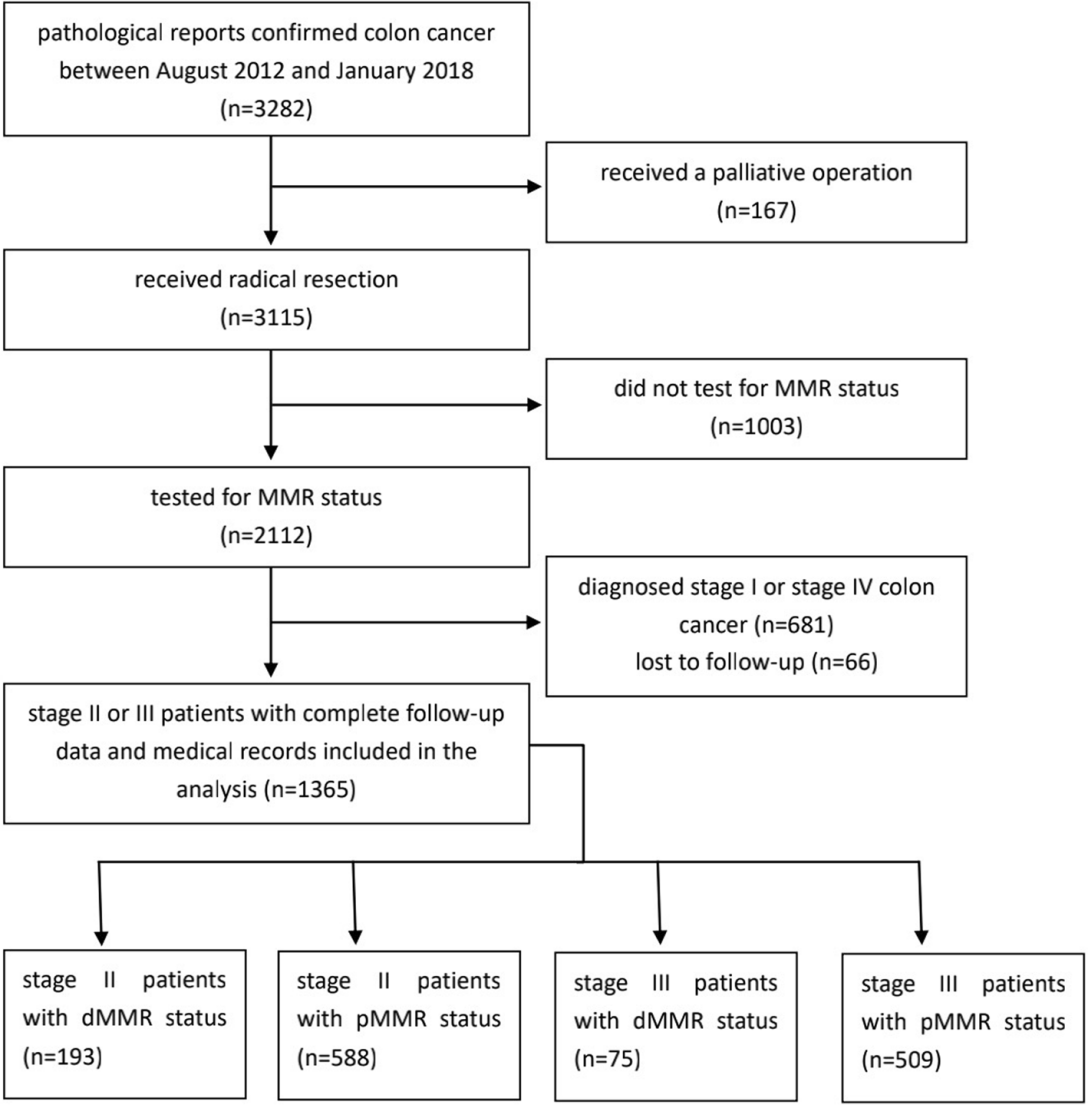
Flow diagrams of the study population

The exclusion criteria were as follows: 1. age younger than 18 years, 2. unknown MMR status, 3. diagnosis of a familial neoplasm, such as familial adenomatous polyposis, Lynch syndrome, or Peutz–Jeghers syndrome, 4. follow-up time of less than 3 years, and 5. death within 1 month postoperatively (Fig. 1).

All patient survival and death data were obtained from the hospital information system, local healthcare security administration and return visit by phone. The survival status of each patient was confirmed again in February 2021. Assessments of the cause and time of death, the chemotherapy regimen administered, time of second cancer occurrence and time of relapse were performed.

### Chemotherapy regimens

Some patients underwent postoperative adjuvant chemotherapy as recommended by their doctors and based on the clinicopathological stage. Chemotherapy regimens and the frequency of delivery were determined during the follow-up and data collection period. There were two main chemotherapy regimens used: FU-based chemotherapy (capecitabine or 5-FU and leucovorin) and oxaliplatin-based chemotherapy (FOLFOX or XELOX).

Stage II patients with one or more of the following characteristics were considered high-risk patients: perineural invasion, pathologic stage T4, vascular invasion, lymphatic infiltration, initial bowel obstruction, tumor perforation, or fewer than 12 excised lymph nodes. These patients were categorized into the high-risk stage II group.

### MMR analysis

MMR status was determined by immunohistochemistry (IHC) in all enrolled patients. dMMR is defined as the deletion of one or more of the four MMR proteins (MLH1, MSH2, MSH6 or PMS2). pMMR is defined by the presence of all four proteins. Tumor MMR expression was analyzed using formalin-fixed, paraffin-embedded tumor samples, and two pathologists jointly determined the MMR status. Supplementary Fig. 1 demonstrates reference IHC staining images of positive and negative nuclear expression for MMR system.

### Propensity score matching

Colon cancer patients with dMMR were found to be older at diagnosis and tended to have proximal colon involvement and a lower tumor stage. To reduce imbalances in the population caused by dMMR patient characteristics, we implemented propensity score matching between the surgery alone and chemotherapy groups. The propensity score was determined via a multivariable Cox regression model. We selected the covariates that could affect survival time for inclusion in the propensity model. The factors considered for inclusion were TNM stage, tumor location, age, sex and chemotherapy. dMMR colon patients were matched with pMMR colon patients at a 1:2 ratio according to a greedy nearest-neighbor matching algorithm without replacement. A caliper width equal to 0.2 of the standard deviation was utilized as the logit of the propensity score.

### Statistical methods

The primary endpoint was OS, which was defined from the date of surgery to the date of death from any cause. DFS was the secondary endpoint. DFS was determined from the surgery date to the date of local or metastatic recurrence or death from any cause, whichever occurred first.

Classified variables were analyzed using a χ^2^ test. Continuous variables were analyzed using a t test. All significance tests were two-sided, and *P* values less than 0.05 were considered statistically significant. All statistical analyses were performed with SPSS software version 23 (SPSS Inc., Chicago, IL, USA).

## Results

### Population characteristics

Overall, 1365 stage II or III colon cancer patients were included in this study. The median follow-up time was 47.0 months, and 26.6% of the patients died. A total of 572 (41.9%) patients received radical resection surgery alone, with a median follow-up time of 48.0 months. A total of 793 (58.1%) patients received adjuvant chemotherapy postoperatively, with a median follow-up time of 47.0 months. In the chemotherapy group, 474 (59.8%) patients received FOLFOX, 109 (13.75%) patients received XELOX, 87 (11.0%) patients received FOLFIRI, and 123 (15.5%) patients received FU alone. 781 (57.2%) patients had stage II disease at diagnosis, and the other 584 (42.8%) patients had stage III disease. The median age at diagnosis was 64.0 years (range, 21 to 89 years), and latest Chinese cancer statistics show that the age groups of more than 60 years accrued most cancer deaths. So, we performed statistical analyses with 60 years old as the boundary. The baseline characteristics of the patients who received surgery alone and those who received chemotherapy are shown in Table 1.

**Table 1:** Demographic and clinical characteristics of all included patients

Patient characteristics	Total (No. %)	Surgery-alone group (No. %)	Chemotherapy group (No. %)	***p***
**TNM**				< 0.001
II	781 (57.2%)	367 (64.2%)	414 (52.2%)	
III	584 (42.8%)	205 (35.8%)	379 (47.8%)	
**Sex**				0.165
Male	746 (54.7%)	300 (52.4%)	446 (56.2%)	
Female	619 (45.3%)	272 (47.6%)	347 (43.8%)	
**Age**				< 0.001
≤60	497 (36.4%)	134 (23.4%)	363 (45.8%)	
> 60	868 (63.6%)	438 (76.6%)	430 (54.2%)	
**T**				0.168
1	3 (0.2%)	1 (0.2%)	2 (0.3%)	
2	20 (1.5%)	5 (0.9%)	15 (1.9%)	
3	1080 (79.1%)	467 (81.6%)	613 (77.3%)	
4	262 (19.2%)	99 (17.3%)	163 (20.6%)	
**N**				< 0.001
0	781 (57.2%)	367 (64.2%)	414 (52.2%)	
1	438 (32.1%)	151 (26.4%)	287 (36.2%)	
2	146 (10.7%)	54 (9.4%)	92 (11.6%)	
**MMR**				0.355
dMMR	268 (19.6%)	119 (20.8%)	149 (18.8%)	
pMMR	1097 (80.4%)	453 (79.2%)	644 (81.2%)	
**Differentiation**				0.264
Low grade (1/2)	902 (66.1%)	388 (67.8%)	514 (64.8%)	
High grade (3/4)	463 (33.9%)	184 (32.2%)	279 (35.2%)	
**Location**				0.313
Proximal colon	632 (46.3%)	274 (47.9%)	358 (45.1%)	
Distal colon	733 (53.7%)	298 (52.1%)	435 (54.9%)	
**Operation**				0.003
Open surgery	888 (65.1%)	398 (69.6%)	490 (61.8%)	
Laparoscopic	477 (34.9%)	174 (30.4%)	303 (38.2%)	
**Total**	1365	572 (41.9%)	793 (58.1%)	

### MMR status as a prognostic marker for the whole cohort

**Fig. 2:**
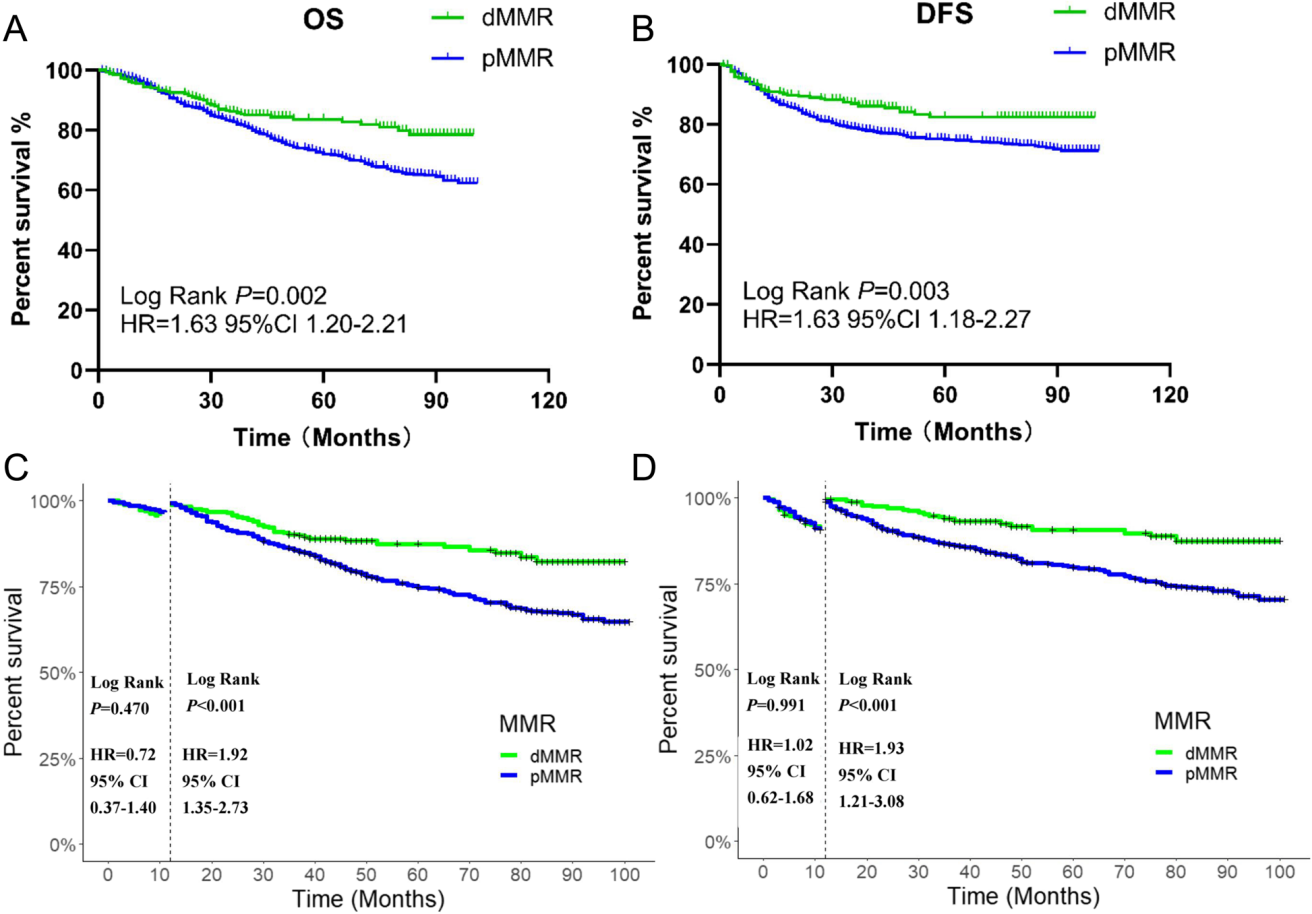
Kaplan-Meier estimates of overall survival (OS) and disease-free survival (DFS) outcomes among all patients. (**a**) OS of all included patients; (**b**) DFS of all included patients; (**c**) Landmark analysis of the OS of all included patients; (**d**) Landmark analysis of the DFS of all included patients

In the whole population, MMR status was a significant prognostic marker. Among all included patients, dMMR status was associated with better OS (*p* = 0.002; Fig. 2a) and DFS outcomes (*p* = 0.003; Fig. 2b). We also observed that the Kaplan–Meier survival curves of the two groups were close within 12 months after surgery. This phenomenon may indicate that MMR status is associated with different prognoses at different periods. Hence, we performed landmark analyses to assess survival differences between the first postoperative year and the period after the first postoperative year. There were no significant differences in OS (*p* = 0.350; Fig. 2c) or DFS (*p* = 0.752; Fig. 2d) outcomes of the dMMR patients or pMMR patients throughout the first year after surgery. However, the OS (*p* < 0.001) and DFS outcomes (*p* < 0.001) between these groups were statistically significant from the second postoperative year until the end of follow-up.

**Fig. 3:**
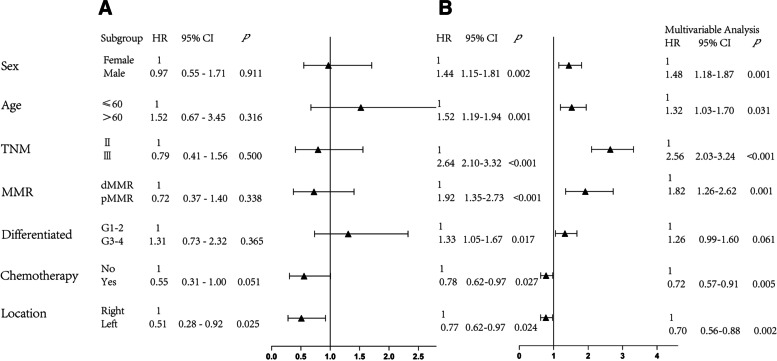
Forest plot of various factors (hazard ratio [HR]) on overall survival (OS). (**a**) in the postoperative first year; (**b**) after the postoperative first year

In the first postoperative year, we found that a distal location led to significantly better OS outcomes (*p* = 0.025, Fig. 3a) Chemotherapy (*p* = 0.051, Fig. 3a), MMR status (*p* = 0.338, Fig. 3a), sex (*p* = 0.911, Fig. 3a) and age (*p* = 0.316, Fig. 3a) had little effect on patient prognosis during this period.

Beyond the first postoperative year, chemotherapy (*p* = 0.0278) and dMMR (*p* < 0.001) were both advantageous factors for OS outcomes (Fig. 3b). In the multivariable Cox analysis, female sex, young age, stage II disease, receiving chemotherapy, dMMR and distal location were associated with better OS.

### MMR status as a prognostic marker for patients with stage II disease

**Fig. 4:**
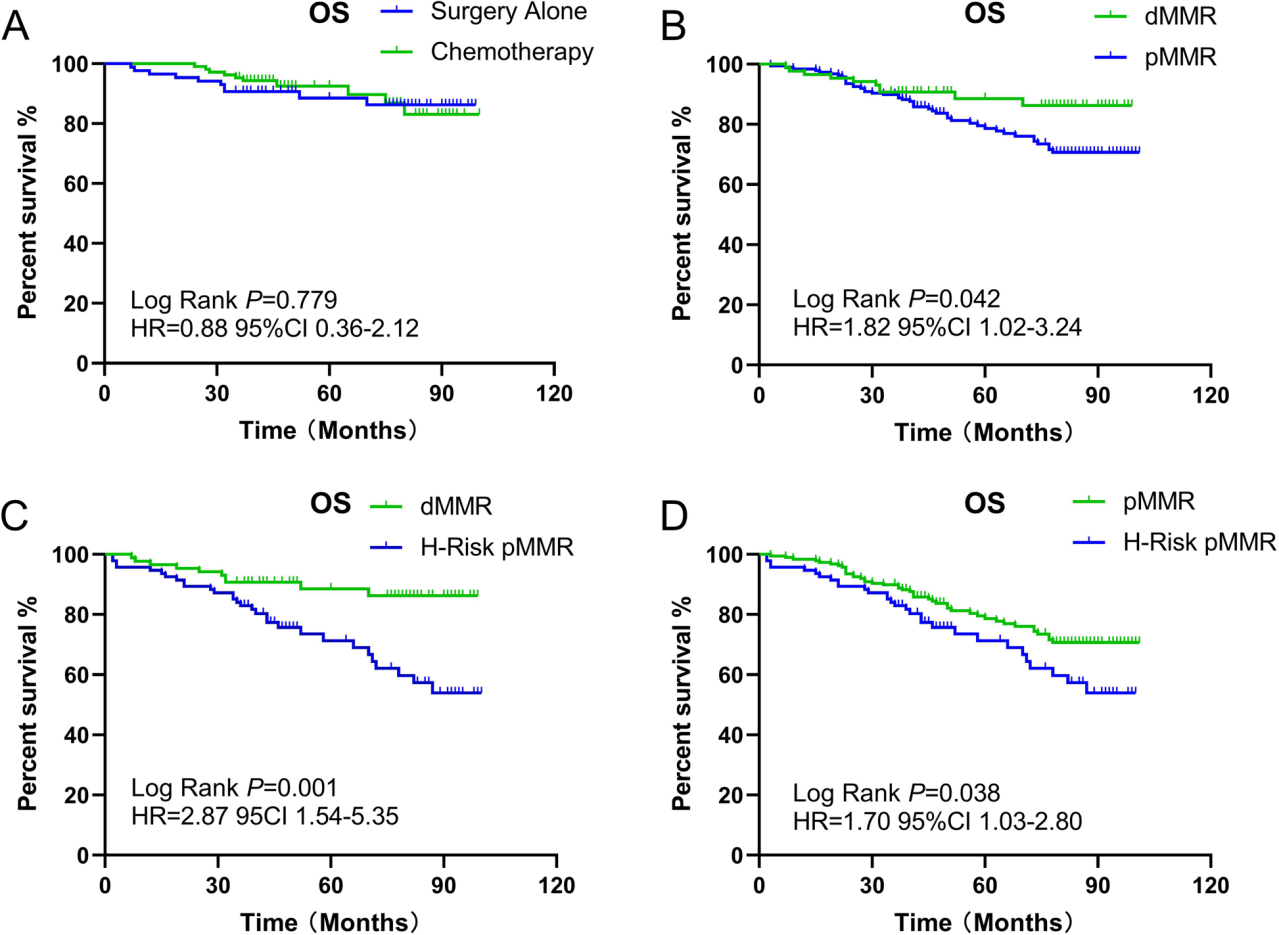
Kaplan-Meier estimates of overall survival (OS) in stage II patients. (**a**) OS of dMMR patients with or without chemotherapy; (**b**) OS of dMMR patients and pMMR patients without high-risk factors;. (**c**) OS of dMMR patients and high-risk pMMR patients; (**d**) OS of pMMR patients with or without high risk factors

Among the stage II colon cancer patients, 193 had dMMR. The OS rate of stage II dMMR colon cancer patients who received adjuvant chemotherapy was 90.7%, and it was 88.4% among those who received radical resection alone. This difference was not statistically significant (*p* = 0.779, Fig. 4a). We specifically analyzed T_4_N_0_M0 patients with dMMR and found that they did not receive any benefit from chemotherapy (log-rank *p* = 0.264).

For the stage II group that did not receive adjuvant chemotherapy, dMMR colon cancer patients had better OS rates than pMMR patients without multiple high-risk factors (88.4% vs. 76.5%, log-rank *p* = 0.042, Fig. 4b). This benefit was also observed in dMMR colon cancer patients compared with pMMR patients with high-risk factors (88.4% vs. 68.1%, log-rank *p* = 0.001, Fig. 4c). In the absence of adjuvant chemotherapy, we also discovered that dMMR patients had better DFS outcomes than pMMR patients with high-risk factors (90.7% vs. 68.1%, log-rank *p* = 0.018). pMMR patients without high-risk factors had better DFS (76.5% vs. 68.1% log-rank *p* = 0.016) and OS (76.5% vs. 68.1% log-rank *p* = 0.038, Fig. 4d) rates than those with high-risk factors.

### MMR status as a prognostic marker for patients with stage III disease

**Fig. 5:**
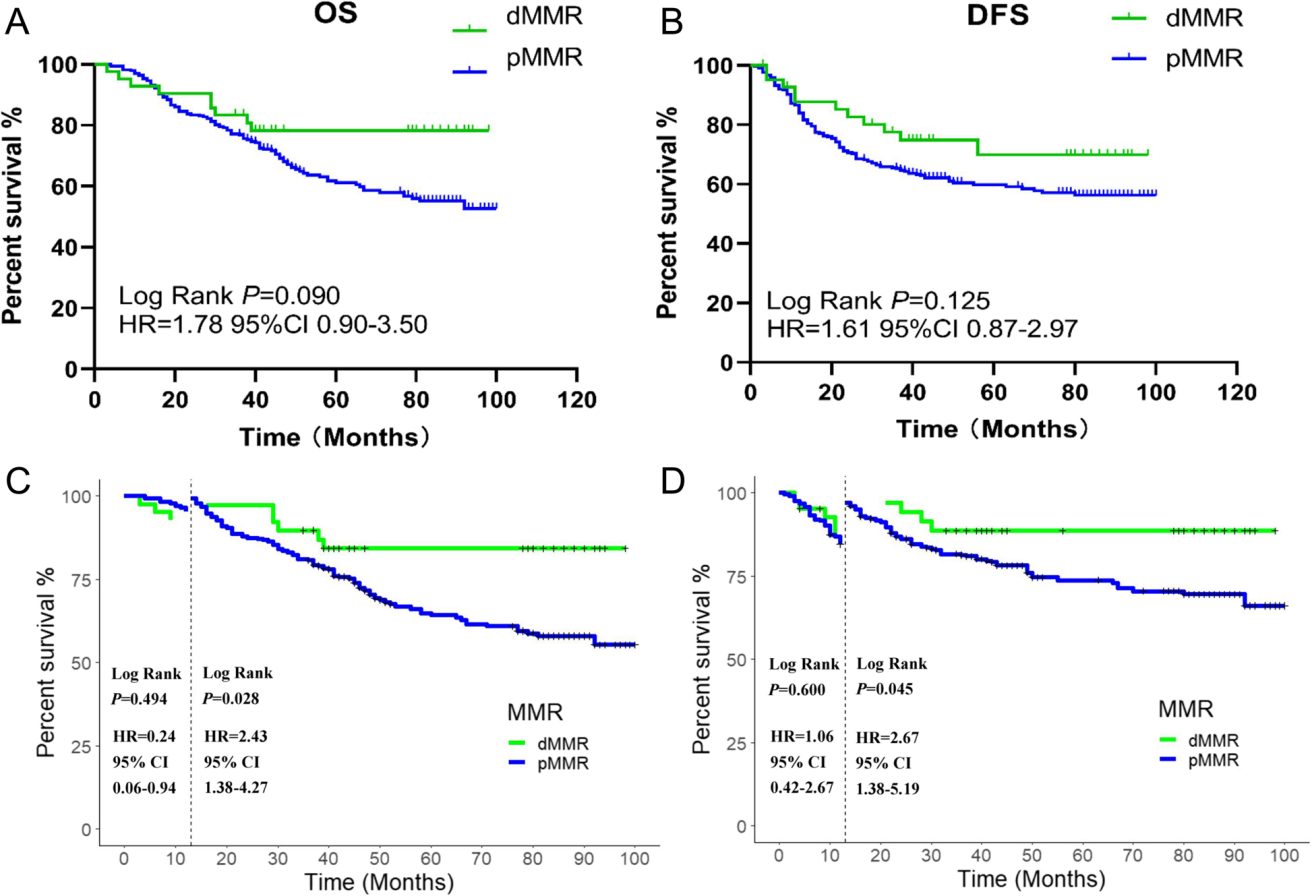
Kaplan-Meier estimates of overall survival (OS) and disease free survival (DFS) in stage III patients with chemotherapy. (**a**) OS of stage III patient; (**b**) DFS of stage III patients; (**c**) Landmark analysis of the OS of stage III patients; (**d**) Landmark analysis of the DFS of stage III patients

Among the patients with stage III colon cancer, 12.8% (*n* = 75) had dMMR. In stage III dMMR colon cancer patients, the group with chemotherapy had a better OS than the counterpart (log-rank *p* = 0.002 S Fig.2). The chemotherapy benefit in the dMMR group was not statistically significant compared with that in the pMMR group in terms of OS (log-rank *p* = 0.090) and DFS (log-rank *p* = 0.125) outcomes. Alternatively, by using landmark analysis to evaluate survival differences 1 year after radical excision, we found an interesting phenomenon similar to that observed in the analysis of all colon cancer patients: during the first year after surgery, there was no statistically significant difference in OS (*p* = 0.494, Fig. 5.) or DFS (*p* = 0.600, Fig. 5) outcomes between the dMMR and pMMR groups.

From the second postoperative year until the end of follow-up, stage III patients with dMMR who received chemotherapy had better OS than stage III patients with pMMR (*p* = 0.028, Fig. 5). This difference was also observed for DFS (*p* = 0.045, Fig. 5).

### Overall survival and disease-free survival after propensity score matching

We used propensity score matching to reduce the imbalances in patient characteristics. After 1:2 propensity score matching, 504 pMMR patients were matched with 263 dMMR patients. In these new cohorts, the standardized differences for all included covariates between dMMR and pMMR patients were all below 0.1 (Table 2).

**Table 2:** Demographic and clinical characteristics of all included patients after propensity score matching

Patient characteristics	Before matching			***p***	After matching			***p***
	Total (No. %)	dMMR (No. %)	pMMR (No. %)		Total (No. %)	dMMR (No. %)	pMMR (No. %)	
**TNM**				< 0.001				0.558
II	781	193 (72.0%)	588 (53.6%)		538 (70.1%)	188 (34.9%)	350 (65.1%)	
III	584	75 (28.0%)	509 (46.4%)		229 (29.9%)	75 (32.8%)	154 (67.2%)	
**Sex**				0.071				0.400
Male	746	136 (50.7%)	610 (55.6%)		404 (52.7%)	133 (32.9%)	271 (67.1%)	
Female	619	132 (49.3%)	487 (44.4%)		363 (47.3%)	130 (35.8%)	233 (64.2%)	
**Age**				0.005				0.777
≤60	497	109 (40.7%)	388 (35.4%)		298 (38.9%)	104 (34.9%)	194 (65.1%)	
> 60	868	159 (59.3%)	709 (64.6%)		469 (61.1%)	159 (33.9%)	310 (66.1%)	
**Chemotherapy**				0.106				0.333
No	572	119 (44.4%)	453 (41.3%)		320 (41.7%)	116 (36.3%)	204 (63.7%)	
Yes	793	149 (55.6%)	644 (58.7%)		447 (58.3%)	147 (32.9%)	300 (67.1%)	
**Location**				< 0.001				0.807
Proximal colon	632	179 (66.8%)	453 (41.3%)		503 (65.6%)	174 (34.6%)	329 (65.4%)	
Distal colon	733	89 (33.2%)	644 (58.7%)		264 (34.4%)	89 (33.7%)	175 (66.3%)	
**Total**	1365	268 (19.6%)	1097 (80.4%)		767	263 (34.3%)	504 (65.7%)	

There was also no statistically significant difference between the dMMR and pMMR groups in terms of OS (*p* = 0.062) and DFS (*p* = 0.750) outcomes during the postoperative first year for all included colon cancer patients. However, significant differences in OS (*p* = 0.005) and DFS (*p* = 0.009) outcomes were found after the first postoperative year.

Likewise, among stage III patients treated with chemotherapy, after propensity score matching, the difference between dMMR and pMMR patients in the first year after radical resection was not remarkable. After the first year of surgery, the benefit of dMMR for OS was not very significant *(p* = 0.050).

### Role of chemotherapy in the elderly population

This cohort study identified 224 (16.4%) patients aged ≥75 years who underwent curative resection. In this group, 187 patients refused chemotherapy for various reasons. Of the 106 (47.3%) stage III patients, 23 (21.7%) received adjuvant chemotherapy after surgery.

The median age was 62 years (range: 21–74 years) in the non-elderly group and 79 years (range: 75–89 years) in the elderly group. Compared with younger patients, a higher proportion of elderly patients had tumors in the proximal colon (45.0% vs. 52.7% *p* = 0.036), refused chemotherapy (82.5% vs. 33.7% *p* < 0.001), and underwent laparoscopic surgery (80.8% vs. 62.0%; *p* < 0.001). There was no difference in the ratio of stage II to stage III disease between these two groups (41.9% vs. 47.3%; *p* = 0.133).

**Fig. 6:**
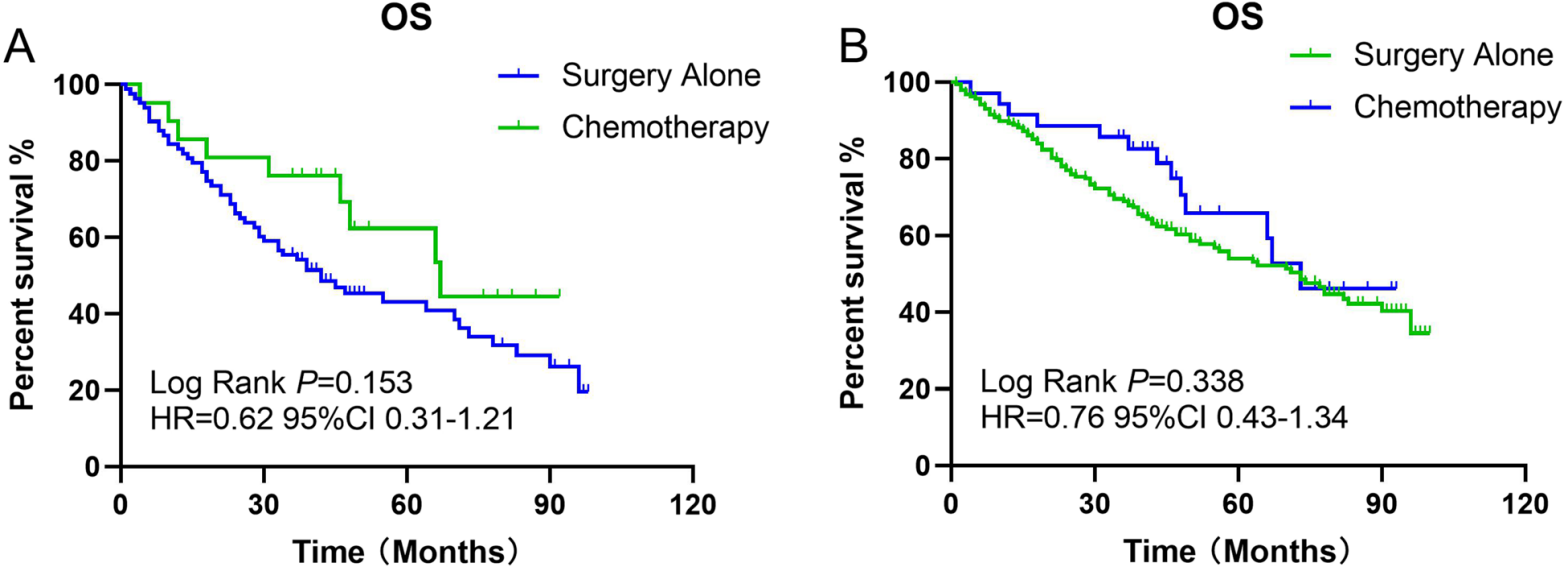
Kaplan-Meier estimates of overall survival (OS) in eldely patients. (**a**) OS of stage III elderly patients with or without chemotherapy; (**b**) OS of all elderly patients with or without chemotherapy

The OS rate of all patients who received chemotherapy was 77.3%, and the OS rate of stage III patients who received chemotherapy was 65.2%. In the older group, the OS rate of all included patients who received chemotherapy was 62.2%, and that for stage III patients who received chemotherapy was 56.5%. Kaplan–Meier analysis of OS revealed that younger stage III patients benefited from adjuvant chemotherapy (log-rank *p* = 0.002, Fig. 6a), but the older group did not (log-rank *p* = 0.153). For all older patients, this benefit still cannot be observed (log-rank *p* = 0.338, Fig. 6b). Although this benefit of chemotherapy for OS was significant in all including patients (log-rank *p* = 0.001).

## Discussion

MMR status, as a prognostic marker of CRC prognosis and a predictive marker of the response to Fu-based chemotherapy [27], can help clinicians and patients better select therapeutic schedules, especially for stage II and III patients [28]. We focused on the prognostic effect of MMR status in stage II and III CRC patients through the rigorous screening of target groups. The dMMR system leads to the increase mutation rates of frameshift and missense mutations in microsatellites, which result in a genetic instability [29]. Existing research has proven that IHC and PCR testing are equally effective at confirming MMR/MSI status [30]. This study mainly investigated whether MMR status affected prognosis during different postoperative periods in patients receiving chemotherapy and whether older patients can benefit from chemotherapy.

Among all stage II patients who did not receive chemotherapy, the dMMR group had a better prognosis than the pMMR group with or without high-risk factors. Stage II patients with low-risk and stage II patients with high-risk patients can benefit from Fu-based and oxaliplatin-based chemotherapy, respectively. This finding has also been demonstrated in previously published studies [31]. However, these studies have rarely separated dMMR patients from pMMR patients with or without high-risk factors for subsequent comparison. In the Kaplan–Meier survival analysis, dMMR was found to be a significant factor associated with better prognosis in stage II patients. The good prognosis of dMMR stage II colon cancer patients, compared with that of pMMR patients, get few benefits from adjuvant chemotherapy. These results again highlight that dMMR is a protective factor for good prognosis in stage II colon cancer patients and suggest the need to select postoperative chemotherapy regimens according to appropriate prognostic and predictive markers.

Since the MOSAIC study was released, postoperative oxaliplatin-based adjuvant chemotherapy therapy has been the standard treatment therapeutic schedule for stage III colon cancer patients worldwide [32]. Therefore, we conducted a separate analysis of the effect of dMMR on the prognosis of stage III patients undergoing chemotherapy. The 1-year DFS rate of stage III dMMR patients who received chemotherapy was 88.1%, while for pMMR patients, it was 86.6%; this difference was not statistically significant (*p =* 0.560). This phenomenon was also observed in the 1-year OS rate (dMMR 92.9% vs. pMMR 96.4% *p =* 0.494). In patients treated with adjuvant chemotherapy, dMMR status was associated with improved DFS and OS outcomes after the first postoperative year.

Colon cancer patients with dMMR were found to be older at diagnosis and tended to have proximal colon involvement and a lower tumor stage [33]. However, given the limitations related to the observational nature of this study and the size of the dMMR group, the baseline populations of dMMR and pMMR patients were imbalanced. It is unknown whether the distinction before and after 1 year is caused by the difference in patient composition or the characteristics of dMMR itself. We alleviated these potential compositional biases by using propensity score matching, which led to well-balanced covariate distributions between these two groups. After matching, the dMMR group had an obvious prognostic advantage in OS and DFS outcomes compared with the pMMR group in all patients from 1 year after surgery to the end of follow-up. This significant difference was consistent before and after matching in stage III patients who received chemotherapy in the subgroup analysis. In contrast, this difference did not exist during the first postoperative year in any of the enrolled patients or in stage III patients treated with chemotherapy. These indicate that MMR status was a significant prognostic marker after the first postoperative year.

The prognosis of the dMMR group has been conventionally considered to be superior to that of the pMMR group among those with stage III disease [34, 35]. However, our findings suggest that this superiority is not consistent across all postoperative stages. The metabolites of 5-FU are incorporated into RNA and DNA and inhibit thymidylate synthase resulting in depletion of dTTP and incorporation in DNA of uracil [36]. The proficiency functional of MMR in FU processing should ensure that the drug is efficiently removed from DNA before it can interfere with essential DNA metabolic processes, such as transcription [37]. Dysfunction in the MMR system causes base excision repair, a process that is less affected by the deoxy-ribonucleotide triphosphate disequilibrium induced by 5-FU, to become the only way to repair mismatched base pairs [38]. Huabin Hu et al. found that a 6-month oxaliplatin-based chemotherapy regimen was superior to a 3-month oxaliplatin-based chemotherapy regimen because long-term adjuvant chemotherapy distorts the secondary DNA structure, which cannot be discerned by MMR complexes [39–41]. These results indicate that dMMR patients can obtain benefit from long-term chemotherapy. However, a part of dMMR patients died during the chemotherapy period, potentially because dMMR confers no significant survival advantage at 1 year postoperatively. The protective effect of dMMR on prognosis for stage III patients was observed only after long-term chemotherapy. This could explain why the prognostic advantage of dMMR did not appear until 1 year after surgery. This may also suggest that starting chemotherapy as early as possible can confer benefits to dMMR patients more quickly. However, this conclusion needs to be confirmed by large clinical cohort studies. The lack of a prognostic advantage for the dMMR group during the first postoperative year indicates that the frequency of review should not be reduced during this phase.

In the elderly patient group, we did not observe a benefit from chemotherapy overall or among stage III patients. This finding is consistent with those of Kawamura Hidetaka, Bergquist John R. and Sanoff, Hanna K [22, 23, 42]. This phenomenon could be due to a variety of factors. Previous studies have revealed that the prognosis of Asian patients with colon cancer is better than that of non-Asian patients [23, 42], which may influence who should receive chemotherapy. Increasing age is also closely related to a higher incidence of complications and a decline in the proportion of deaths attributed to colon cancer [43]. The percentage of people who die from colon cancer declines with increasing patient age; simultaneously, the proportion of deaths from complications increases [43]. Moreover, the postoperative mortality rate of patients aged ≥80 years is nearly 10 times that of patients in other age groups [44]. Increasing evidence from Europe has proven that the high risk of postoperative mortality from comorbidities can endure beyond 1 year after surgery [17]. Furthermore, older patients may be at higher risk for cardiovascular events and septic complications, which is related to worse postoperative long-term survival outcomes [45]. Most studies have focused only on complications during the first 30 postoperative days; nevertheless, mortality caused by postoperative complications has been shown to persist 1 year after surgery [46].

Few multicenter studies with large populations have been conducted in East Asia. Multicenter studies, strict inclusion criteria and large numbers of patients make the conclusions of these studies more representative. Propensity score matching was used to unify the baseline characteristics of the study population and to make the different groups more comparable.

Although this retrospective study explored the influence of MMR status and age on the prognosis of stage II and III patients, there are inevitably some drawbacks. First, the small sample size of patients with dMMR made it difficult to divide patients into sufficient groups for analysis. Some patients could not tolerate the ADRs of oxaliplatin-based chemotherapy regimens, they eventually received FOLFIRI chemotherapy. The effect of this chemotherapy regimen on outcomes is unknown, further data collection and analysis of the treatment outcomes of the dMMR patients are pending. Secondly, all hospitals used the IHC method to detect MMR during the early stage due to the lack of standardized kits and the time-consuming nature of PCR. Therefore, this study lacks PCR data to verify the IHC results which would result in a lower detected MMR mutation rate than actuality. About 5 to 11% of MSI cases will not show MMR protein loss, because missense mutations in the MMR gene can lead to functional inactivation of the protein without affecting its stability and antigenicity [47, 48]. Moreover, gene detection analysis is not covered by health insurance, our present study did not include the RAF and RAS statuses in these patients. Approximately 30% ~ 50% of sporadic dMMR cases are associated with BRAF mutations [49, 50].

## Conclusion

The results of this study demonstrate that the prognosis of stage III colon cancer patients with dMMR is not significantly different from that of patients with pMMR during the first postoperative year. The benefit of dMMR for prognosis could be observed from the second postoperative year until the end of follow-up. Thus, we should increase the frequency of patient review to reduce the likelihood of recurrence and non-cancer-related death. This study also demonstrates that elderly patients aged ≥75 with stage II or stage III disease obtain no significant benefit from postoperative chemotherapy, because recurrence and metastasis are not the main causes of death in elderly patients after surgery.

## Supplementary Information

**Additional file 1:** **Figure S1.** Representative typical immunohistochemical staining images of positive and negative nuclear expressions of MLH1, MSH6 and PMS2

**Additional file 2:** **Figure S2.** Kaplan-Meier estimates of overall survival (OS) in stage III dMMR patients with or without chemotherapy

## Data Availability

The datasets used and analyzed during the current study are available from the corresponding author on reasonable request.

## Abbreviations

dMMR: Deficient mismatch repair
OS: Overall survival
DFS: Disease-free survival
pMMR: Proficient mismatch repair
CRC: Colorectal cancer
MMR: Mismatch repair; IHC: immunohistochemistry; MMR: Mismacth repair.
