# Supplementary figures and images for: Effects of deficient mismatch repair on the prognosis of patients with stage II and stage III colon cancer during different postoperative periods
Source: BMC Cancer. 2022 Nov 10;22:1156. doi: 10.1186/s12885-022-10266-3 (PMC9648020; doi:10.1186/s12885-022-10266-3)

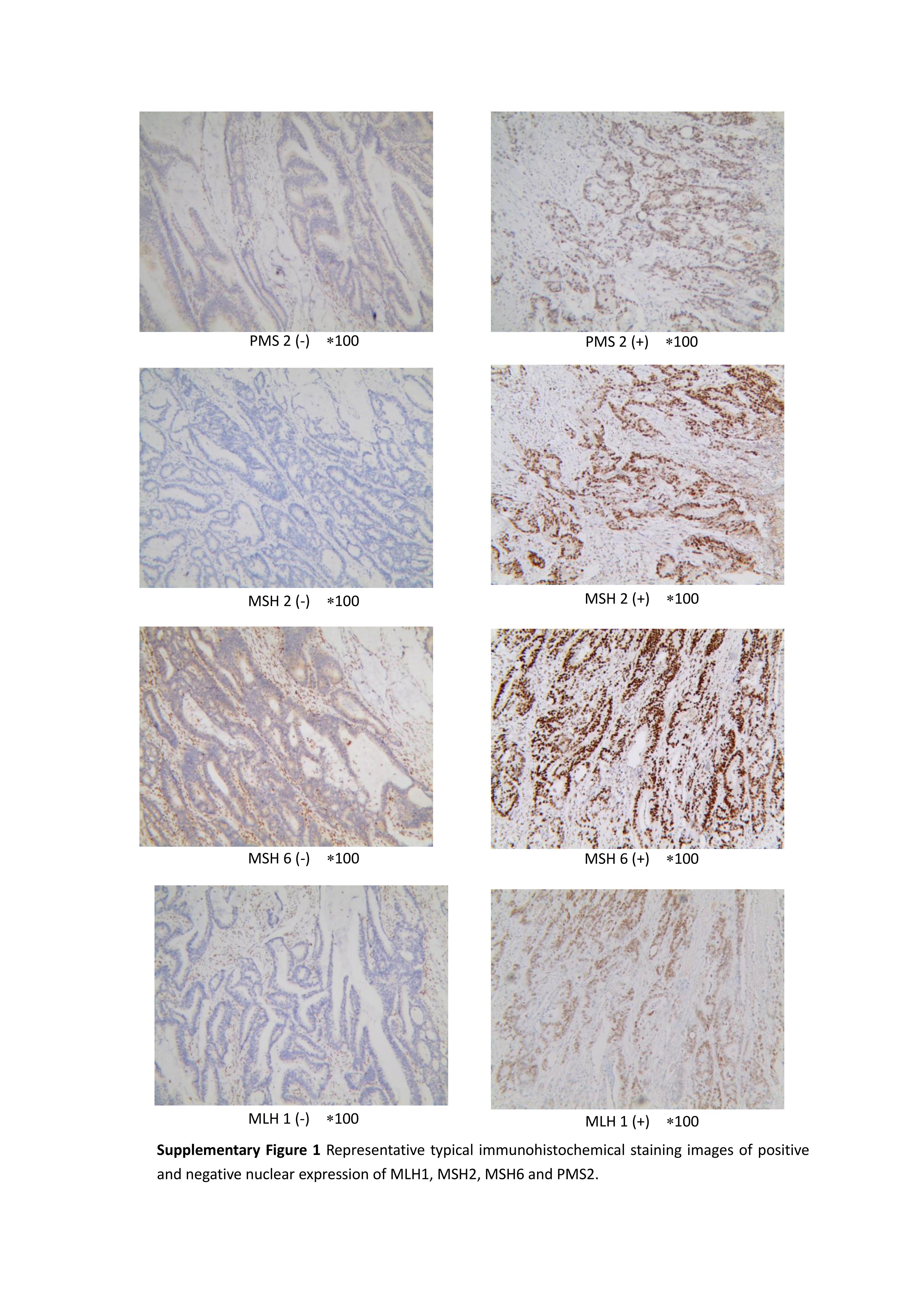

Supplement: Supplementary file 1 — Additional file 1: Figure S1. Representative typical immunohistochemical staining images of positive and negative nuclear expressions of MLH1, MSH6 and PMS2 [file 12885_2022_10266_MOESM1_ESM.jpg]

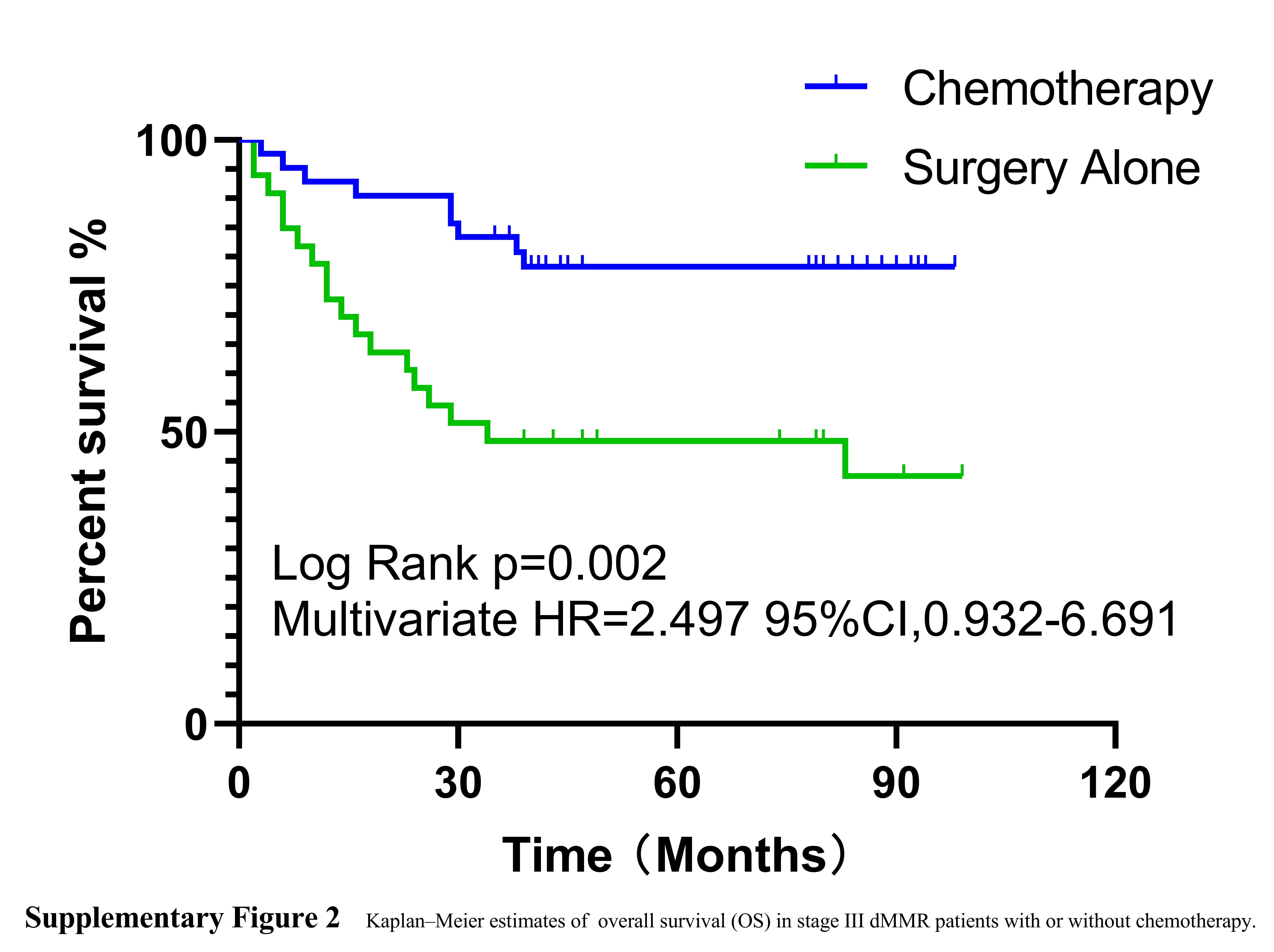

Supplement: Supplementary file 2 — Additional file 2: Figure S2. Kaplan-Meier estimates of overall survival (OS) in stage III dMMR patients with or without chemotherapy [file 12885_2022_10266_MOESM2_ESM.tif]
